# Incidental Prenatal Discovery of Anencephaly via Portable Ultrasounds in a Remote Medical Care Setting

**DOI:** 10.7759/cureus.96998

**Published:** 2025-11-16

**Authors:** Colby V Spongberg, Elaine Webber, Rilee Schmidt, James Hanna, Dan Ayeko, Becky A Slater

**Affiliations:** 1 College of Medicine, Touro College of Osteopathic Medicine, Great Falls, USA; 2 Surgery, Kapchorwa Hospital, Kapchorwa, UGA; 3 Emergency Medicine, Touro College of Osteopathic Medicine, Great Falls, USA

**Keywords:** anencephaly, folic acid deficiency, maternal malnutrition, neural tube defect, polyhydramnios, portable ultrasound, prenatal care, resource-limited settings, uganda

## Abstract

Anencephaly is a fatal neural tube defect often linked to folic acid deficiency and undetected in resource-limited settings. We report a case in rural Uganda where portable ultrasound revealed anencephaly with polyhydramnios in a patient who has been pregnant six times, with one term delivery, four preterm deliveries, and no abortions, and currently has one living child (G6P1401). Early detection of this anomaly enabled surgical planning and maternal counseling before stillbirth. This case highlights the impact of malnutrition and the importance of portable ultrasound use to improve prenatal care in underserved regions of the world.

## Introduction

Anencephaly and other nutrient deficiency-related birth defects are prevalent in socioeconomically challenged regions such as Uganda and are rarely appreciated prior to childbirth due to limited technological resources, to the detriment of patients and caregivers [1-3]. Anencephaly is a lethal cranial neural tube defect that manifests with an absent or underdeveloped cranium, forebrain, and cerebellum. A major cause of anencephaly is folic acid (vitamin B9) deficiency in early pregnancy. This results in polyhydramnios due to a defective fetal swallowing center [4]. Anencephaly was the second most common neural tube defect observed in the Ugandan population. While approximately half of the anencephaly cases were liveborn, all died soon after birth [2].

The device used for this study was the Butterfly IQ+ ultrasound probe used with an Apple iPad Pro (Apple Inc., California, USA).

## Case presentation

**Figure 1:**
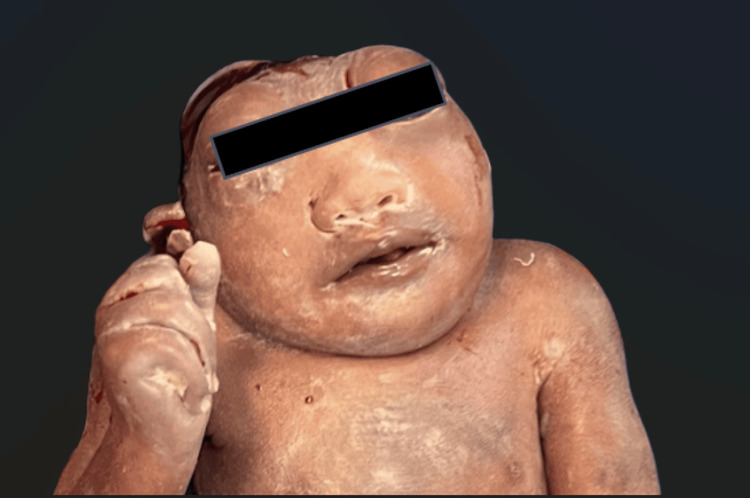
Postnatal anencephaly fetus delivered via a cesarean section.

In July of 2024, in the Kapchorwa District of Uganda, a case of polyhydramnios with potential anencephaly was discovered incidentally during the course of routine diagnostic imaging by an emergency medicine-trained physician using a portable ultrasound device of a multiparous mother. In discussions with local providers, it was noted that the mother was classified as G6P1401 at approximately 25 weeks of gestational age, who had not received prenatal care prior to this visit. Several of the patient’s previous pregnancies resulted in birth defects attributed to malnutrition that is common to the geographic region where the patient resides, with only one child surviving past infancy. The fetus was alive during the initial encounter with an ultrasound-observed heart rate in the 100 bpm range. The heart rate fell within 90 bpm on the subsequent day, suggesting fetal distress. The decision was made to deliver via cesarean section after failure of misoprostol. Cesarean delivery proceeded without complications, the fetus was deceased at birth (Figure 1), and the postnatal course was uneventful. This was a rare instance where an issue was discovered prior to delivery, providing an opportunity for counselling of the patient and preparation of the local surgical team prior to delivery of the stillborn child. The cesarean section mitigated potential issues from a likely complicated natural birth attempt and subsequent interventions, as well as the unexpected emotional distress to the mother.

Informed consent for the use and publication of the image in Figure 1 has been duly obtained in accordance with ethical and regulatory standards. 

## Discussion

Anencephaly is characterized by failure of neural tube closure, causing a calvarium defect with a lack of brain tissue [5]. The devastating congenital defect is incompatible with life and very few survive beyond one year of age [6,7]. A variety of environmental and nutritional factors have been implicated in the pathogenesis, including sporadic genetic mutations, malnutrition, and toxic exposure [8-10]. Although awareness of the issue is rising, the global prevalence of this anomaly is still significant [11]. The use of ultrasound allows anencephaly to be accurately diagnosed in the first trimester, around 12-14 weeks of gestational age [12,13]. Families are faced with significant stress and long-term psychological sequelae after experiencing stillbirth, including depression, anxiety, and post-traumatic stress disorder [14-17]. Early detection allows healthcare practitioners to counsel the patient and their families prior to birth, providing the opportunity to decrease the emotional stress of stillbirth [18,19]. 

This case highlights the prenatal care issues of the area, including but not limited to malnutrition and the lack of diagnostic imaging. It anecdotally supports the fielding of portable ultrasound devices as low-cost adjuncts in resource-limited areas, in support of prenatal care and the expansion of the study of complications surrounding such care.

The use of portable ultrasound devices serves as an efficient tool for diagnostic imaging in the course of prenatal care in remote environments, to highlight and mitigate the impact of potentially traumatic situations arising from the delivery of children with previously detectable birth defects. Consistent and early use of such devices presents an opportunity to improve patient outcomes, as well as a medium for further deepening of the study of, and appreciation for, the need for adequate prenatal care and nutrition in underserved environments [20-22].

## Conclusions

This case illustrates the value of portable ultrasound devices as accessible and effective tools for prenatal screening in resource-limited settings. Their deployment can aid in the early detection of fatal anomalies such as anencephaly, improve clinical preparedness, and reduce the trauma associated with unforeseen birth complications. Furthermore, it highlights the need to address maternal nutrition and expand diagnostic infrastructure in underserved regions to improve maternal and neonatal health outcomes. This case also exemplifies core principles of osteopathic medicine, particularly the holistic approach to patient care and the emphasis on prevention, education, and community health. By identifying anencephaly early, the care team was able to counsel the mother, prepare the surgical team, and mitigate potential physical and emotional trauma, reflecting the osteopathic focus on patient-centered care. This case highlights how the osteopathic principles of holistic care, preventive medicine, and community empowerment can be applied globally to enhance maternal and fetal health outcomes.
